# The global and regional burden of genital ulcer disease due to herpes simplex virus: a natural history modelling study

**DOI:** 10.1136/bmjgh-2019-001875

**Published:** 2020-03-08

**Authors:** Katharine Jane Looker, Christine Johnston, Nicky J Welton, Charlotte James, Peter Vickerman, Katherine M E Turner, Marie-Claude Boily, Sami L Gottlieb

**Affiliations:** 1 Population Health Sciences, Bristol Medical School, University of Bristol, Bristol, UK; 2 Department of Medicine, University of Washington, Seattle, Washington, USA; 3 Virology Research Clinic, Seattle, Washington, USA; 4 Vaccine and Infectious Diseases Division, Fred Hutchinson Cancer Research Center, Seattle, Washington, USA; 5 Bristol Veterinary School, University of Bristol, Bristol, UK; 6 Department of Infectious Disease Epidemiology, Imperial College London, London, UK; 7 Department of Reproductive Health and Research, World Health Organization, Geneve, Switzerland

**Keywords:** epidemiology, infections, diseases, disorders, injuries

## Abstract

**Introduction:**

Herpes simplex virus (HSV) infection can cause painful, recurrent genital ulcer disease (GUD), which can have a substantial impact on sexual and reproductive health. HSV-related GUD is most often due to HSV type 2 (HSV-2), but may also be due to genital HSV type 1 (HSV-1), which has less frequent recurrent episodes than HSV-2. The global burden of GUD has never been quantified. Here we present the first global and regional estimates of GUD due to HSV-1 and HSV-2 among women and men aged 15–49 years old.

**Methods:**

We developed a natural history model reflecting the clinical course of GUD following HSV-2 and genital HSV-1 infection, informed by a literature search for data on model parameters. We considered both diagnosed and undiagnosed symptomatic infection. This model was then applied to existing infection estimates and population sizes for 2016. A sensitivity analysis was carried out varying the assumptions made.

**Results:**

We estimated that 187 million people aged 15–49 years had at least one episode of HSV-related GUD globally in 2016: 5.0% of the world’s population. Of these, 178 million (95% of those with HSV-related GUD) had HSV-2 compared with 9 million (5%) with HSV-1. GUD burden was highest in Africa, and approximately double in women compared with men. Altogether there were an estimated 8 billion person-days spent with HSV-related GUD globally in 2016, with 99% of days due to HSV-2. Taking into account parameter uncertainty, the percentage with at least one episode of HSV-related GUD ranged from 3.2% to 7.9% (120–296 million). However, the estimates were sensitive to the model assumptions.

**Conclusion:**

Our study represents a first attempt to quantify the global burden of HSV-related GUD, which is large. New interventions such as HSV vaccines, antivirals or microbicides have the potential to improve the quality of life of millions of people worldwide.

**Key questions**

**What is already known?**

- Herpes simplex virus (HSV) type 2 infections are abundant globally and can cause painful, recurrent genital ulcer disease (GUD), the natural history of which has been well documented.
- Genital HSV type 1 infection also causes GUD, but recurrences tend to be much less frequent.
- The population burden of GUD, given global estimates of HSV infection and the known natural history of GUD, has never been quantified.

**What are the new findings?**

- Using a natural history model applied to infection estimates and population sizes, we estimated that 187 million people (5.0% of the world’s population) aged 15–49 years had at least one episode of HSV-related GUD globally in 2016, with an estimated 8 billion person-days spent with HSV-related GUD in this year.
- GUD burden was highest in Africa, approximately double in women compared with men, and almost entirely due to HSV type 2.

**What do the new findings imply?**

- The global burden of HSV-related GUD is large and affects all regions of the world, although disproportionately affects certain populations.
- Interventions targeted against HSV, such as new vaccines, antivirals or microbicides, have the potential to improve the quality of life of millions of people worldwide.

## Introduction

Genital herpes is a lifelong sexually transmitted infection caused by herpes simplex virus type 2 (HSV-2) and type 1 (HSV-1) and is characterised by recurrent, self-limited outbreaks of painful genital lesions in a proportion of those infected.^1^ We estimated that in 2016, 491 million people aged 15–49 years had HSV-2 infection, which is almost all sexually transmitted.^2^ HSV-1, although predominately an oral infection, is increasing as a cause of genital herpes in some settings.^3–5^ We estimated that 192 million people had genital HSV-1 infection in 2016.^2^ Symptoms of genital HSV infection include vesicles, ulcers, fissures or other painful lesions on or near the genital skin and mucosa, collectively termed genital ulcer disease (GUD). Although many people acquire genital HSV infection without symptoms, in those who do have GUD, the first episode typically lasts the longest (up to 2–3 weeks in the absence of antiviral therapy) and may be more severe and associated with systemic symptoms.^6^ First-episode GUD is clinically indistinguishable whether caused by HSV-2 or HSV-1 infection.^6^ However, those with HSV-2-related GUD often have subsequent periodic recurrences of GUD over many years, while HSV-1-related GUD seems to recur much less frequently.^7^ GUD recurrences tend to be shorter and less severe than the first episode.^6 7^

Symptomatic HSV infection is associated with psychosocial effects, including low mood, feelings of shame and stigma, fears around transmission, and effects on personal relationships.^8^ There is potential for transmission to sexual partners, and to the neonate during birth, regardless of symptoms, as viral shedding occurs both asymptomatically and symptomatically.^9–11^ Neonatal infection is rare but can be extremely serious, as it carries a high risk of neonatal death or permanent disability.^12^ Current epidemiological evidence suggests that HSV-2 infection also increases susceptibility to HIV infection,^13 14^ and may increase HIV infectiousness in people living with HIV (PLHIV).^13 15 16^ Since GUD is associated with increased quantities of viral shedding, as well as breaches to the protective integrity of the genital skin and mucosa, these transmission risks and cofactor effects may be heightened in the presence of GUD.^17^

The global burden of HSV-related GUD is not well understood due to a lack of dependable, systematic surveillance or prevalence studies in most settings. Case reporting may not be reliable because many symptomatic individuals do not seek care and remain undiagnosed,^18^ and GUD may not be recognised as herpetic by clinicians, since although HSV is the most common cause of GUD globally GUD can have other causes.^19–26^ A more complete understanding of the morbidity associated with HSV infection, and in particular GUD, would help elucidate the full potential public health value of HSV interventions.^27^ Here we present the first global and regional estimates of GUD due to HSV-1 and HSV-2 among women and men aged 15–49 years old using a natural history model reflecting the clinical course of GUD following HSV-2 and genital HSV-1 infection with existing HSV-2 and genital HSV-1 infection estimates for 2016.^2^

## Methods

**Figure 1:**
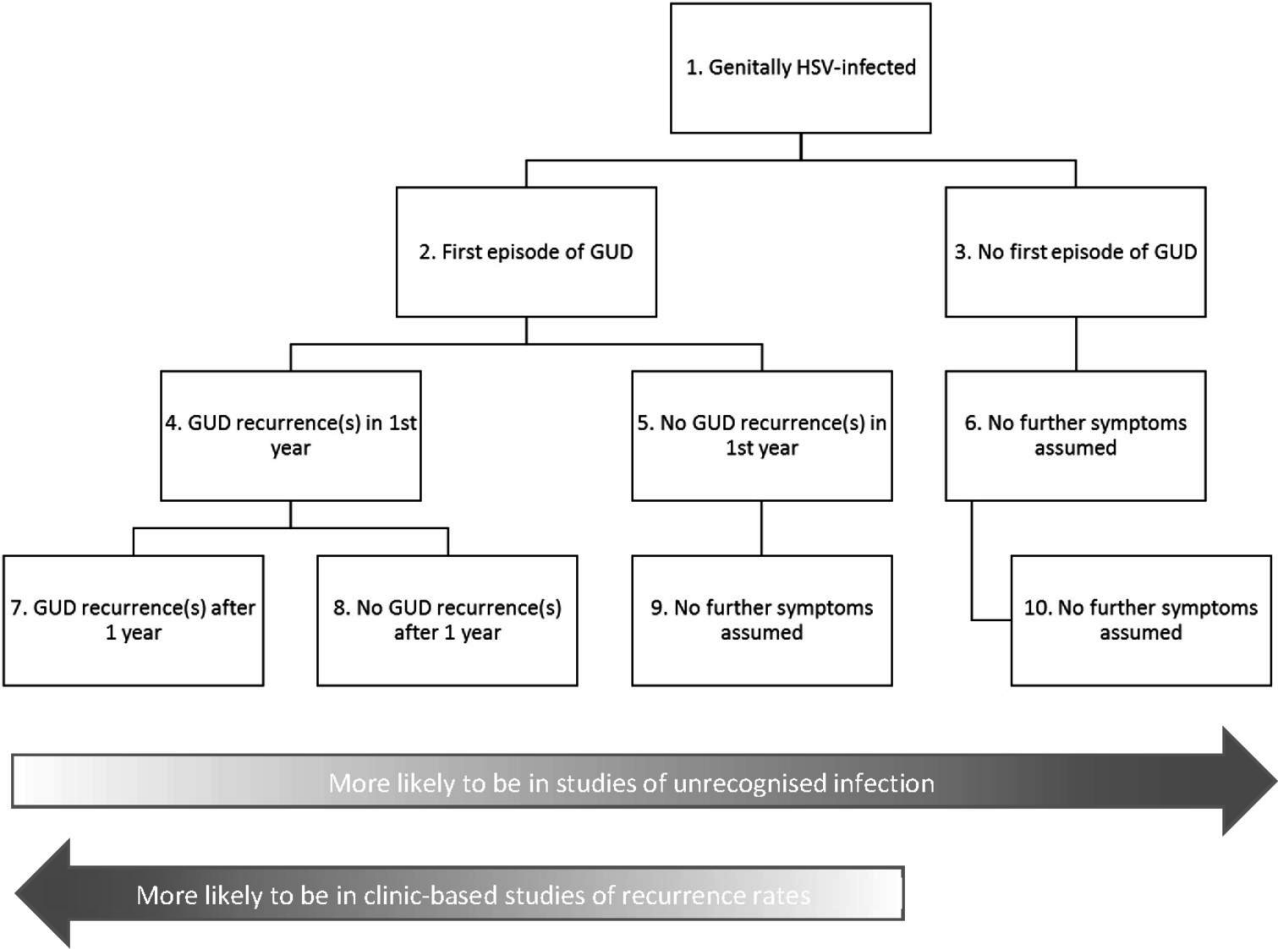
Flow chart showing the different possible GUD states relevant to the GUD estimates. Please refer to table 1 for definitions of terms, and online supplementary table A1 for information on how these states map to the natural history parameters used in the GUD burden estimates. Note that this is a simplified representation for the purposes of the estimation process. GUD, genital ulcer disease; HSV, herpes simplex virus.

We aimed to estimate GUD due to HSV-2 or genital HSV-1 infection. Two different measures of GUD burden most relevant for public health were derived separately for HSV-2 and genital HSV-1 infection: (1) the percentage and the number of people aged 15–49 years with any GUD in a given year; and (2) the total number of person-days with GUD among individuals aged 15–49 years old in a given year. The clinical course of infection varies between individuals (eg, not all individuals genitally infected with HSV experience a first GUD episode; and in those who do, not all have subsequent recurrences) and between HSV-1 and HSV-2 (HSV-2 recurrences are more frequent than HSV-1 recurrences). A flow chart showing the different possible states/clinical courses for GUD relevant to the GUD estimates is shown in figure 1. The GUD burden measures therefore required considering and summing the percentage and time spent in each relevant possible GUD state. GUD burden estimates were generated using natural history parameter estimates for genital HSV-1 and HSV-2 infection (obtained from a literature search) with published HSV-2 and genital HSV-1 infection estimates^2^ and population sizes for 2016.^28^

10.1136/bmjgh-2019-001875.supp1 Supplementary data

**Table 1:** Definitions used

Term	Definition used
Genital ulcer disease (GUD)	Genital lesions, ulcers or vesicles due to either herpes simplex virus (HSV) type 2 or genital HSV type 1.
Recently acquired infection	Up to 1 year since infection was acquired.
Established infection	More than 1 year since infection was acquired.
First episode	First noted GUD symptoms.
Recurrence	GUD symptoms subsequent to the first episode.

To align with our previous WHO estimates of the annual incidence and prevalence of HSV-2 and genital HSV-1 infection,^2^ we defined an individual as having ‘recently-acquired’ infection if up to 1 year since HSV infection acquisition, and as having an ‘established infection’ if more than 1 year since HSV acquisition. We defined a first episode of GUD as the first noted GUD symptoms, regardless of whether those symptoms occur before or after HSV seroconversion (ie, development of new HSV type-specific antibodies on infection). A first episode is generally thought to occur within the first year since HSV infection acquisition, and for the purpose of these estimates we assumed that the first episode always occurs within the first year. An occurrence of GUD symptoms subsequent to the first episode was termed a recurrence. For a full list of the definitions used, please refer to table 1.

### Natural history model

#### GUD due to HSV-2 infection

##### Any GUD in a given year

The percentage of people with at least one episode of GUD due to HSV-2 in a given year at age *a* (as a percentage of the total population) can be expressed by the following:

$$\left[I\left(a\right)*P_{first}\right]+\left[F\left(a\right)*P_{{recur}_{\tau §amp;gt;1}}\right]$$

where:

$I\left(a\right)$is the percentage of the population with recently acquired HSV-2 infection (among all individuals, not just those with HSV-2 infection).

$P_{first}$is the percentage of individuals with recently acquired infection (τ≤1 year since infection) who have a first episode of GUD.

$F\left(a\right)$is the percentage of the population with established HSV-2 infection (among all individuals, not just those with HSV-2 infection).

$P_{{recur}_{\tau §amp;gt;1}}$is the percentage of individuals with established infection (τ>1 year since infection) who have one or more GUD recurrences in a year after the first year of infection.

As shown in online supplementary table A1, estimates for $I\left(a\right)$ and $F\left(a\right)$ were taken from the 2016 WHO estimates for HSV-2 infection prevalence and incidence, and $P_{first}$ from studies that followed people for new HSV infection as measured by seroconversion, and then evaluated those people for symptoms. For $P_{{recur}_{\tau §amp;gt;1}}$, estimates were informed by two types of studies:

- Clinic-based studies that recruited individuals with a diagnosed first episode due to HSV-2 and measured the percentage of individuals with at least one recurrence during follow-up.
- Studies that recruited individuals who were HSV-2 seropositive but without a history of recognised genital herpes and observed how many experienced documented GUD during follow-up.

Thus, to incorporate these two types of data, we expanded the equation to evaluate the proportion with a recurrence among diagnosed individuals, θ=1, and undiagnosed individuals, θ=0:

$$\begin{array}{l} \left[I\left(a\right)*P_{first}\right]+\left[F\left(a\right)*([P_{\theta =1}*P_{recur_{\tau >1,\theta =1}}\right]+\\ \left[P_{\theta =0}*P_{recur_{\tau >1,\theta =0}}\right])] \end{array}$$

where:

$P_{\theta =1}$is the percentage of individuals with HSV-2 infection who are diagnosed.

$P_{\theta =0}$is the percentage of individuals with HSV-2 infection who are undiagnosed (equal to ${1-P}_{\theta =1}$).

To get $P_{\theta =1}$, we used the estimate of the proportion of HSV-2 infections that are diagnosed from the largest and most recent national population-based survey for the USA (the country where the majority of the natural history studies were conducted), which is the National Health and Nutrition Examination Survey 2007–2010^18^ (online supplementary table A1). Although the proportion of infected individuals who are diagnosed will vary widely between countries, this estimate is used here solely to determine the proportion of individuals to which clinic-based parameter data apply versus the proportion to which parameter data from studies of individuals with unrecognised infection apply.

##### Number of person-days with HSV GUD

The mean number of days with GUD due to HSV-2 in a given year at age *a* can be expressed by the following:

$$\begin{array}{l} {}[I(a)*P_{first}*T_{first_{\tau \leq 1}}]+[I(a)*([P_{\theta =1}*T_{recur_{\tau \leq 1,\theta =1}}]+\\ {}[P_{\theta =0}*T_{recur_{\tau \leq 1,\theta =0}}])]+[\sum_{x=1}^{x=9}I(a-x)*([P_{\theta =1}*T_{recur_{1<\tau \leq 10,\theta =1}}]+\\ {}[P_{\theta =0}*T_{recur_{1<\tau \leq 10,\theta =0}}])]+[\sum_{x=10}^{x=a-15}I(a-x)*([P_{\theta =1}*T_{recur_{\tau >10,\theta =1}}]+\\ {}[P_{\theta =0}*T_{recur_{\tau >10,\theta =0}}])] \end{array}$$

where:

${T_{first}}_{\tau \leq 1}$is the number of GUD days per person with recently acquired infection (τ≤1) experiencing a first episode.

$T_{{recur}_{\tau \leq 1}}$is the number of GUD days per person with recently acquired infection (τ≤1) due to all recurrences in the first year (averaged over all those with recently acquired infection, including those without recurrences), among diagnosed individuals, θ=1, and undiagnosed individuals, θ=0.

$T_{{recur}_{1§amp;lt;\tau \leq 10}}$is the number of GUD days per person 1<τ≤10 years following infection due to all recurrences in a year (averaged over all those with established infection, including those without recurrences), among diagnosed individuals, θ=1, and undiagnosed individuals, θ=0.

$T_{{recur}_{\tau §amp;gt;10}}$is the number of GUD days per person τ>10 years following infection due to all recurrences in a year (averaged over all those with established infection, including those without recurrences), among diagnosed individuals, θ=1, and undiagnosed individuals, θ=0.

Natural history parameters for those with diagnosed infection were obtained from clinic-based studies, while natural history parameters for those with undiagnosed infection were obtained from studies of individuals with unrecognised infection (online supplementary table A1).

#### GUD due to HSV-1 infection

Genital HSV-1 estimates were derived in a similar fashion to those done for HSV-2, except that recurrences were limited to the first 5 years since infection because there is a low recurrence rate during the first 5 years of infection and no data available for the past 5 years. In addition, there were no identified studies of recurrences in those with unrecognised infection, and the percentage with genital HSV-1 infection who are diagnosed is unknown: only *P*_*first*_ can be estimated. The equations are as follows:

$$\left[I\left(a\right)*P_{first}\right]+\left[\sum_{x=1}^{x=4}I\left(a-x\right)*{P_{first}*P}_{{recur}_{\tau §amp;gt;1│first}}\right]$$

$$\begin{array}{l} \left[I\left(a\right)*P_{first}*T_{first_{\tau \leq 1}}\right]+\left[I\left(a\right)*P_{first}*T_{recur_{\tau \leq 1}|first}\right]+\\ \left[\sum_{x=1}^{x=4}I\left(a-x\right)*P_{first}*T_{recur_{\tau >1|first}}\right] \end{array}$$

where:

$P_{{recur}_{\tau §amp;gt;1│first}}$is the percentage of individuals with established infection (τ>1 year since infection) and who had a first episode who have one or more GUD recurrences in a year after the first year of infection.

$T_{{recur}_{\tau \leq 1│first}}$is the number of GUD days per person with recently acquired infection (τ≤1) due to all recurrences in the first year among those who had a first episode (including those without recurrences).

$T_{{recur}_{\tau §amp;gt;1│first}}$is the number of GUD days per person τ>1 years following infection due to all recurrences in a year among those who had a first episode (including those without recurrences).

For more details on how all the equations were derived, see online supplementary appendix.

GUD estimates were derived separately by HSV type, WHO region (Americas, Africa, Eastern Mediterranean, Europe, South-East Asia and Western Pacific), age and sex, and summed to generate global estimates. We assumed the total HSV-related GUD burden was equal to the sum of the burden for each of HSV-1 and HSV-2.

### Literature search and pooling

Natural history parameters were informed by a PubMed literature search of English-language titles reporting on longitudinal studies (date of search: 06 November 2017). Data were extracted from studies which met specific inclusion criteria to ensure data were comparable. Natural history data obtained from the literature search were then standardised as follows: data on episode duration ($T_{{first}_{\tau \leq 1}}$ and $D_{{recur}_{\tau }}$) reported as medians were converted to means, and data on recurrence frequency ($N_{{recur}_{\tau }}$) were annualised if necessary, and medians converted to means. Data for each parameter (separately for individuals with diagnosed vs undiagnosed infection, where applicable) were then pooled in Stata V.13.1 using meta-analysis assuming a random-effects model. All relevant data were pooled for each parameter in question: we did not perform separate pooling by sex or geographical location for example, nor explore the effect of covariates on pooled estimates, due to limited available data. Log study estimates and the SE of each log estimate were used for pooling, and the resultant pooled estimates converted back to the natural scale. All natural history data were in the absence of antivirals, with the exception of a few studies where antiviral use was unknown. A further two studies used in the pooling reported episodic therapy,^9 29^ but neither of these provided data on the duration of a first episode or recurrence.

Our GUD estimates follow the Guidelines for Accurate and Transparent Health Estimates Reporting (GATHER).^30 31^ A completed GATHER checklist is given in the online supplementary appendix. Full details of the literature search and pooling are given in the online supplementary appendix. For a full list of the parameter values and 95% CI used in the uncertainty analysis, see online supplementary table A1.^2 7 9 18 32–84^

### Sensitivity analysis

We identified three areas of uncertainty which might particularly influence the GUD estimates. First is uncertainty around how long people continue to experience HSV-2 recurrences, since recurrence rates for the past 10 years were only informed by two studies (online supplementary table A1). Second is uncertainty around the percentage of the HSV-2-infected population to which recurrence rates as measured in clinic-based studies versus studies of unrecognised infection apply. Clinic rates may be biased towards those with more severe disease, and studies of unrecognised infection miss those who have already been diagnosed with HSV-related GUD, which may vary by setting. Third is uncertainty around the percentage of the HSV-2 infected population that truly has HSV-related symptoms, as even in prospective studies of seroconversion with assessment of symptoms, identification of GUD is dependent on how thoroughly and frequently study participants are counselled, followed up and assessed. In a clinical trial of both women and men with the largest sample size in terms of number of HSV-2 seroconversions, and which rigorously searched for and evaluated those with possible HSV-related symptoms, 35.5% had documented first-episode GUD at any time around or following seroconversion.^36^ A sensitivity analysis was carried out for the HSV-2 GUD burden estimates, (1) limiting recurrences to the first 10 years since acquisition of HSV-2 infection, (2) applying recurrence natural history parameters from studies of those with unrecognised infection to all those with HSV-2 infection, (3) applying recurrence natural history parameters obtained from clinic studies to the maximum percentage with GUD symptoms in the first year as measured in a rigorous clinical trial^36^ (recurrence natural history parameters from studies of those with unrecognised infection not used), and (4) applying recurrence natural history parameters obtained from clinic studies to the percentage with GUD symptoms in the first year as measured in all studies ($P_{first}$), and recurrence natural history parameters from studies of those with unrecognised infection to all remaining infected individuals.

### Uncertainty bounds

We derived 95% uncertainty intervals (UI) of the percentage and the number of people aged 15–49 years with any GUD and the total number of person-days with GUD among those aged 15–49 years old using a Monte Carlo sampling method to sample all natural history parameters in online supplementary table A1 1000 times in Excel. Pooled log estimates and the SE of the pooled log estimate obtained from the meta-analysis were used for sampling and a normal distribution was assumed. Uncertainty in the HSV-1 and HSV-2 infection estimates was also incorporated by concurrently sampling the log force of infection using the log fitted force of infection and the SE of the log fitted force of infection by sex and WHO region, again assuming a normal distribution. For full details, please see the corresponding paper.^2^ The 95% UI was based on the 2.5 and 97.5 percentiles from the set of 1000 generated GUD burden estimates.

### Patient and public involvement

Patients and the public were not involved in this study.

## Results

### Number and percentage of people with any GUD

**Figure 2:**
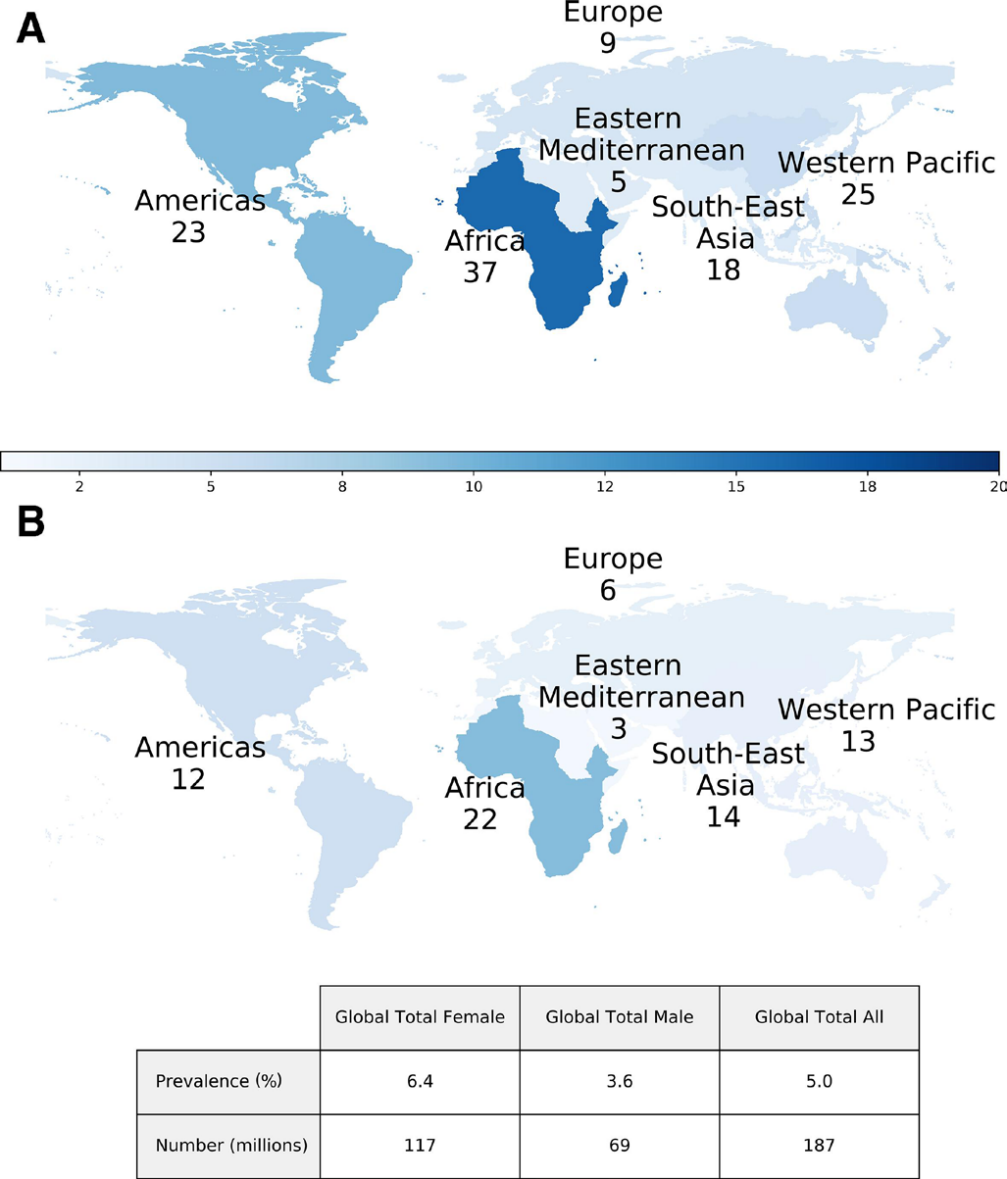
Estimated number of people (in millions) aged 15–49 years with any GUD due to HSV in 2016, among women (A) and men (B). Percentage of people with any GUD due to HSV in 2016 shown by the colour gradient. GUD, genital ulcer disease; HSV, herpes simplex virus.

We estimated that 187 million people aged 15–49 years had at least one episode of HSV-related GUD globally in 2016, equivalent to 5.0% of the world’s population (figure 2). Of these, 178 million (95% of those with HSV-related GUD) had at least one episode of GUD due to HSV-2 in 2016 (4.8%) vs 9 million (5%) due to genital HSV-1 (0.2%) (table 2). The burden of GUD (any HSV type) was highest in Africa (59 million), followed by the Western Pacific (39 million), the Americas (35 million) and South-East Asia (32 million); the burden of GUD due to HSV-1, however, was highest in the Americas (4 million).

**Table 2:** Global and regional estimates of the number (in millions) and percentage of people aged 15-49 years with any GUD due to HSV-2 and HSV-1 in 2016, by age, sex and WHO region

WHO region	Sex	Age (years)							
		15–19	20–24	25–29	30–34	35–39	40–44	45–49	All ages
HSV-2									
Americas	Female	1.0	1.9	2.7	3.3	3.7	4.0	4.3	20.9
		2.8%	5.1%	7.2%	9.2%	11.1%	12.8%	14.4%	8.7%
	Male	0.5	0.9	1.3	1.6	1.8	2.0	2.1	10.1
		1.3%	2.4%	3.4%	4.5%	5.4%	6.4%	7.3%	4.2%
Africa	Female	3.9	5.6	6.3	6.3	5.8	5.0	4.2	37.1
		7.7%	12.7%	16.4%	19.1%	21.1%	22.6%	23.6%	15.8%
	Male	1.8	2.8	3.4	3.7	3.6	3.3	2.9	21.5
		3.5%	6.3%	8.9%	11.2%	13.3%	15.3%	17.1%	9.2%
Eastern Mediterranean	Female	0.3	0.5	0.7	0.8	0.8	0.8	0.8	4.6
		0.9%	1.6%	2.4%	3.1%	3.8%	4.5%	5.2%	2.8%
	Male	0.1	0.2	0.3	0.3	0.3	0.3	0.3	1.8
		0.3%	0.6%	0.9%	1.1%	1.4%	1.7%	1.9%	1.0%
Europe	Female	0.3	0.5	0.9	1.2	1.5	1.7	1.9	8.0
		1.1%	2.0%	2.9%	3.8%	4.7%	5.5%	6.3%	3.9%
	Male	0.1	0.3	0.5	0.6	0.7	0.8	0.9	4.0
		0.5%	1.0%	1.4%	1.9%	2.3%	2.8%	3.2%	1.9%
South-East Asia	Female	0.7	1.3	1.8	2.2	2.5	2.6	2.7	13.9
		1.1%	2.0%	2.9%	3.8%	4.6%	5.5%	6.3%	3.5%
	Male	0.7	1.3	1.8	2.2	2.5	2.6	2.7	13.9
		0.8%	1.5%	2.2%	2.9%	3.5%	4.2%	4.8%	2.6%
Western Pacific	Female	0.8	1.7	3.2	3.6	3.9	5.3	6.5	25.1
		1.5%	2.8%	4.0%	5.2%	6.3%	7.4%	8.5%	5.3%
	Male	0.4	0.9	1.6	1.9	2.0	2.8	3.4	13.0
		0.7%	1.3%	1.9%	2.5%	3.1%	3.7%	4.3%	2.6%
Global	Female	7.2	11.9	16.0	18.1	18.8	20.1	21.1	113.2
		2.6%	4.2%	5.5%	6.7%	7.7%	8.6%	9.4%	6.2%
	Male	3.7	6.4	8.9	10.3	11.0	11.9	12.3	64.5
		1.2%	2.1%	2.9%	3.7%	4.4%	4.9%	5.4%	3.4%
	Both	10.9	18.3	24.9	28.4	29.8	32.0	33.4	177.7
		1.9%	3.1%	4.1%	5.1%	6.0%	6.7%	7.4%	4.8%
HSV-1									
Americas	Female	0.3	0.4	0.4	0.3	0.2	0.2	0.2	2.1
		0.9%	1.2%	1.0%	0.9%	0.7%	0.6%	0.5%	0.8%
	Male	0.3	0.4	0.4	0.3	0.2	0.2	0.2	2.0
		0.8%	1.1%	1.0%	0.9%	0.8%	0.7%	0.6%	0.8%
Africa	Female	0.0	0.0	0.0	0.0	0.0	0.0	0.0	0.0
		0.0%	0.0%	0.0%	0.0%	0.0%	0.0%	0.0%	0.0%
	Male	0.0	0.0	0.0	0.0	0.0	0.0	0.0	0.0
		0.0%	0.0%	0.0%	0.0%	0.0%	0.0%	0.0%	0.0%
Eastern Mediterranean	Female	0.3	0.3	0.2	0.1	0.0	0.0	0.0	0.8
		0.9%	0.9%	0.5%	0.3%	0.2%	0.1%	0.1%	0.5%
	Male	0.3	0.3	0.2	0.1	0.0	0.0	0.0	0.9
		0.9%	0.9%	0.5%	0.3%	0.2%	0.1%	0.1%	0.5%
Europe	Female	0.2	0.3	0.2	0.1	0.1	0.1	0.0	1.0
		0.9%	1.0%	0.6%	0.4%	0.3%	0.2%	0.1%	0.4%
	Male	0.2	0.4	0.3	0.3	0.2	0.2	0.2	1.8
		0.9%	1.2%	1.0%	0.9%	0.8%	0.6%	0.5%	0.8%
South-East Asia	Female	0.0	0.0	0.0	0.0	0.0	0.0	0.0	0.1
		0.1%	0.0%	0.0%	0.0%	0.0%	0.0%	0.0%	0.0%
	Male	0.1	0.0	0.0	0.0	0.0	0.0	0.0	0.1
		0.1%	0.0%	0.0%	0.0%	0.0%	0.0%	0.0%	0.0%
Western Pacific	Female	0.0	0.0	0.0	0.0	0.0	0.0	0.0	0.1
		0.3%	0.2%	0.1%	0.0%	0.0%	0.0%	0.0%	0.1%
	Male	0.1	0.1	0.0	0.0	0.0	0.0	0.0	0.2
		0.1%	0.1%	0.0%	0.0%	0.0%	0.0%	0.0%	0.0%
Global	Female	1.0	1.1	0.8	0.5	0.4	0.3	0.2	4.3
		0.4%	0.4%	0.3%	0.2%	0.2%	0.1%	0.1%	0.2%
	Male	1.0	1.1	0.9	0.7	0.5	0.4	0.3	5.0
		0.3%	0.4%	0.3%	0.2%	0.2%	0.2%	0.1%	0.3%
	Both	2.0	2.2	1.7	1.2	0.9	0.7	0.5	9.2
		0.3%	0.4%	0.3%	0.2%	0.2%	0.1%	0.1%	0.2%
HSV-1 and HSV-2									
Global	Female	8.2	13.0	16.8	18.6	19.2	20.4	21.3	117.5
		2.9%	4.6%	5.7%	6.9%	7.9%	8.7%	9.5%	6.4%
	Male	4.7	7.6	9.8	11.0	11.5	12.3	12.7	69.4
		1.6%	2.5%	3.2%	3.9%	4.6%	5.1%	5.6%	3.6%
	Both	12.8	20.6	26.6	29.6	30.7	32.7	33.9	186.9
		2.2%	3.5%	4.4%	5.4%	6.2%	6.9%	7.5%	5.0%

GUD, genital ulcer disease; HSV-1, herpes simplex virus type 1; HSV-2, herpes simplex virus type 2.

The burden of GUD due to HSV-2 infection was approximately double in women compared with men due to higher HSV-2 prevalence in women (table 2). The burden of GUD due to HSV-1 infection was more evenly distributed between the sexes. The burden of GUD increased with age for HSV-2, reflecting increased prevalence with age. The burden of GUD due to genital HSV-1, however, was highest among those aged 20–24 years and declined thereafter, due to the assumption that recurrences were limited to the first 5 years since infection.

### Person-days with GUD

We estimated that there were 8300 million person-days spent with GUD globally in 2016 (assuming an absence of treatment), the vast majority of which were due to HSV-2 (table 3). Genital HSV-1 infection contributed relatively few person-days of GUD (100 million person-days), due to the fact that the recurrence rate was low for genital HSV-1 infection and limited to the first 5 years since infection (table 3 and online supplementary table A1). We estimated that the total number of first episodes in 2016 was approximately twice as high for HSV-2 compared with genital HSV-1 (5.0 million vs 2.4 million, respectively), whereas the number of recurrences was markedly higher for HSV-2 than genital HSV-1 (959 million vs 4.3 million) (online supplementary table A2).

**Table 3:** Global and regional estimates of GUD person-days (in millions) due to HSV-2 and HSV-1 among the total population aged 15-49 years in 2016, by age, sex and WHO region

WHO region	Sex	Age (years)							
		15–19	20–24	25–29	30–34	35–39	40–44	45–49	All ages
HSV-2									
Americas	Female	47	85	121	151	172	187	199	963
	Male	22	41	59	73	83	91	98	466
Africa	Female	183	254	293	300	276	239	202	1747
	Male	84	125	155	170	168	155	135	993
Eastern Mediterranean	Female	12	21	31	38	39	37	36	213
	Male	4	8	12	14	15	15	15	84
Europe	Female	12	24	42	56	67	79	89	369
	Male	6	13	21	28	33	39	44	185
South-East Asia	Female	42	73	104	130	145	154	158	805
	Male	34	59	83	103	114	121	125	640
Western Pacific	Female	37	78	144	165	181	248	303	1155
	Male	20	41	74	85	93	129	156	598
Global	Female	333	534	734	839	879	943	987	5251
	Male	171	287	404	474	508	550	573	2966
	Both	504	821	1139	1313	1387	1493	1560	8217
HSV-1									
Americas	Female	5	4	3	3	2	2	1	21
	Male	4	4	3	3	2	2	2	21
Africa	Female	0.1	0.0	0.0	0.0	0.0	0.0	0.0	0.1
	Male	0.1	0.0	0.0	0.0	0.0	0.0	0.0	0.1
Eastern Mediterranean	Female	4	2	1.2	0.7	0.3	0.2	0.1	8
	Male	4	2	1.3	0.7	0.4	0.2	0.1	9
Europe	Female	3	2	2	1	0.7	0.4	0.3	9
	Male	3	3	3	3	2	2	1	18
South-East Asia	Female	0.6	0.1	0.0	0.0	0.0	0.0	0.0	0.8
	Male	0.7	0.1	0.0	0.0	0.0	0.0	0.0	0.8
Western Pacific	Female	2	1	0.3	0.1	0.0	0.0	0.0	3
	Male	1	0.3	0.1	0.0	0.0	0.0	0.0	1
Global	Female	14	9	7	5	3	2	2	42
	Male	13	10	8	6	5	4	3	50
	Both	27	19	15	11	8	6	5	92
HSV-1 and HSV-2									
Global	Female	347	544	741	844	883	945	989	5293
	Male	184	297	412	480	513	554	576	3016
	Both	531	841	1153	1324	1395	1499	1565	8309

GUD, genital ulcer disease; HSV-1, herpes simplex virus type 1; HSV-2, herpes simplex virus type 2.

We estimated that individuals with HSV-2 infection experienced on average 16 days with GUD in 2016 (ie, averaged over all those with HSV-2 infection whether asymptomatic or symptomatic). HSV-2 infected individuals with GUD in 2016 (ie, those with symptoms), meanwhile, experienced on average 46 days with GUD. For genital HSV-1 infection, 0.8 days were spent with GUD during 2016 on average among all infected individuals, while infected individuals with GUD in 2016 experienced on average 10 days with GUD.

### Sensitivity analysis

The GUD burden estimates are sensitive to the assumptions made. If HSV-2 recurrences are limited to the first 10 years since infection, the percentage with any GUD due to HSV-2 would be lowest at 2.1% (79 million) (online supplementary table A3). If we assume that studies of those individuals with unrecognised infection represent everyone with HSV-2 infection in terms of recurrence rates and duration, then an estimate of 143 million (3.8%) would experience GUD. A similar percentage (4.1%) would experience GUD if we apply rates from clinic populations to the estimated maximum percentage with symptoms in the first year. However, if we apply rates from clinic populations to the average percentage with symptoms in the first year, and rates from studies of those with unrecognised infection to everyone else, an estimate of 5.4% (201 million) would experience GUD.

### Uncertainty bounds

Taking into account uncertainty in HSV infection rates and GUD natural history, the 95% UI for the percentage of people aged 15–49 years with at least one episode of HSV-related GUD globally in 2016 was 3.0%–7.7% for HSV-2, equivalent to between 112 and 289 million affected people, and 0.1%–0.4% for HSV-1, equivalent to 5–16 million people (table 4). The 95% UI for the person-days spent with GUD globally in 2016 was 5500–14 000 million person-days for HSV-2 and 60–500 million person-days for HSV-1. Altogether, 120–296 million people were estimated to have had at least one episode of HSV-related GUD globally in 2016 due to either HSV type, or between 3.2% and 7.9% of the population, equivalent to 5600–14 300 million person-days of disease.

**Table 4:** 95% uncertainty intervals for the percentage and number (in millions) of people aged 15-49 years with any GUD due to HSV-2 and HSV-1 in 2016, and the number of GUD person-days (in millions) due to HSV-2 and HSV-1 among the total population aged 15–49 years in 2016, by sex and WHO region

WHO region	Sex	With any GUD (in %)	With any GUD (n) (in millions)	GUD person-days (in millions)
HSV-2				
Americas	Female	5.2–13.7	12.5–33.0	614–1627
	Male	2.3–7.2	5.6–17.4	265–835
Africa	Female	9.5–24.3	22.3–56.9	1123–2865
	Male	5.4–15.2	12.5–35.5	593–1678
Eastern Mediterranean	Female	1.0–6.7	1.7–11.2	82–541
	Male	0.2–5.5	0.3–9.9	15–471
Europe	Female	1.5–8.4	3.2–17.5	172–857
	Male	0.8–4.6	1.7–9.7	84–447
South-East Asia	Female	1.3–8.5	6.6–42.5	320–1989
	Male	0.8–8.0	4.2–42.4	193–2055
Western Pacific	Female	2.7–9.7	12.7–46.5	625–2227
	Male	1.0–6.7	5.2–33.8	253–1586
Global	Female	3.8–9.8	68.9–180.2	3442–8841
	Male	2.0–5.9	38.8–112.9	1941–5610
	Both	3.0–7.7	111.7–288.6	5507–13 990
HSV-1				
Americas	Female	0.5–1.2	1.2–3.0	13–89
	Male	0.5–1.2	1.2–2.9	13–84
Africa	Female	0.0–0.1	0.0–0.2	0–3
	Male	0.0–0.1	0.0–0.2	0–3
Eastern Mediterranean	Female	0.1–1.1	0.1–1.6	1–36
	Male	0.1–1.1	0.1–1.8	1–38
Europe	Female	0.1–0.8	0.3–1.9	3–45
	Male	0.5–1.2	1.1–2.6	11–78
South-East Asia	Female	0.0–0.1	0.0–0.4	0–6
	Male	0.0–0.1	0.0–0.4	0–7
Western Pacific	Female	0.0–0.5	0.0–2.7	0–31
	Male	0.0–0.5	0.0–2.7	0–31
Global	Female	0.1–0.4	2.4–7.5	26–216
	Male	0.2–0.5	2.9–8.9	32–242
	Both	0.1–0.4	5.3–15.8	58–455
HSV-1 and HSV-2				
Global	Female	4.0–10.1	73.7–184.7	3496–8897
	Male	2.3–6.2	43.7–118.0	1995–5673
	Both	3.2–7.9	120.0–295.6	5577–14 324

GUD, genital ulcer disease; HSV-1, herpes simplex virus type 1; HSV-2, herpes simplex virus type 2.

## Discussion

We estimated that, in 2016, 187 million people aged 15–49 years experienced HSV-related GUD, which was equivalent to 5.0% of the world’s population. Altogether GUD was associated with an estimated 8300 million person-days with disease globally. Taking into account parameter uncertainty, the percentage with at least one episode of HSV-related GUD ranged from 3.2% to 7.9% (120–296 million). Established (prevalent) HSV-2 infection caused the majority of GUD compared with both genital HSV-1 infection and recently acquired (incident) HSV-2 infection, meaning GUD trends predominately reflected HSV-2 prevalence. This reflects the natural history of genital HSV infection, with HSV-2 frequently recurring even years after infection, while recurrences of genital HSV-1 infection are much less frequent. We estimated that 178 million people aged 15–49 years experienced GUD due to HSV-2 in 2016. This estimate varied between 143 and 201 million when assumptions around the recurrence rate and duration were varied, and could be as low as 79 million if recurrences stopped after 10 years. Altogether in 2016 there were an estimated 5.0 million first episodes due to HSV-2, 2.4 million first episodes due to genital HSV-1, 959 million HSV-2 recurrences and 4.3 million genital HSV-1 recurrences. Consistent with HSV-2 epidemiology, GUD burden was highest in Africa, was higher in women versus men, and increased with age. HSV-2 infection has been shown to increase susceptibility to HIV,^14^ and this risk may be even higher in the presence of GUD.^17^ Therefore, the high GUD burden in Africa and in women is particularly concerning as young women in this region are at high risk of acquiring HIV.^85^

### Strengths and limitations

This study represents the first attempt to estimate the burden of GUD due to HSV globally. Our study has several strengths. We used the most recent available WHO estimates of HSV-2 and genital HSV-1 incidence and prevalence and the best available data on natural history parameters from a detailed review of the literature. By reflecting the complex natural history of genital herpes recurrences and incorporating differences between HSV-1 and HSV-2 infection and by time since infection, we generated a useful paradigm for conceptualising the burden of HSV-related GUD. We were able to generate estimates and demonstrate patterns in GUD burden by HSV type, age, sex and WHO region. Importantly, we also considered the contribution of unrecognised infection to disease burden. By highlighting the number of first episodes and recurrences, our estimates can inform the extent to which clinical care is used currently, and the potential for future HSV-2 interventions to impact on the clinical course of infection. There is a lack of dependable, systematic surveillance of GUD or prevalence studies in most settings, and case reporting may not be reliable. Therefore these estimates are a first step in understanding the total burden of GUD, rather than just the limited number of cases seen in clinical care.

Our estimates have some limitations. First, we did not consider the effect of coinfection with HIV on GUD in our estimates. Our literature review identified some GUD natural history data for PLHIV, from which there was some indication that recurrence frequency and duration can be higher in those who are HIV-positive.^73 86–90^ By not incorporating the effect of HIV infection on GUD, it is likely that we have underestimated GUD burden in settings with high HIV prevalence. However, to allow the natural history of GUD to vary by HIV status, we would have had to estimate the degree of coinfection, factoring in the epidemiological association between the two infections due to shared risk factors and biological effects of each infection on the other,^13 91 92^ and considered the effect of CD4 count and antiretroviral therapy status on GUD.^90^ This would have added in complexity and thus uncertainty, and ultimately we erred on the side of underestimating rather than overestimating GUD burden. Our natural history parameters do not account for antiviral use, which may have already led us to overestimate the number of person-days with GUD. Episodic therapy, which is widely used in many countries, has some effect on symptom duration but no effect on the likelihood of subsequent recurrences.^55^ Daily suppressive therapy, meanwhile, is effective at reducing symptoms and recurrence rate, although it is not available in most countries.^93^ Another consideration is that our estimates were done at the WHO regional level: HIV prevalence average for the entire WHO Africa region was 3.9% among 15–49 years in 2018,^94^ but in South Africa for example this figure was 20.4%.^95^ The HIV–HSV-2 interaction is critically important and this issue should be studied in depth in future, dedicated analyses.

Second, the GUD estimates build on published WHO estimates of HSV-1 and HSV-2 infection, meaning the issues and assumptions affecting the infection estimates, including data availability, generalisability and quality, are carried forward to the GUD estimates.^2^ Furthermore, HSV-1 infection estimates were not produced separately by sex for all regions, meaning GUD estimates for HSV-1 may not fully capture differences by sex. However, the infection estimates were informed by systematic reviews to August 2018 and represent the best attempt to quantify HSV-2 and genital HSV-1 prevalence and incidence globally by age and sex. In some countries, such as the USA, an ‘epidemiological transition’ has already occurred whereby rates of oral HSV-1 infection during childhood have declined, and rates of genital HSV-1 infection have increased, due to decreased immunity to HSV-1 on entering adulthood possibly combined with increasing rates of oral sex.^3 96^ In the most recent prospective evaluation of GUD among women with new HSV infection, in North America, 62% of HSV GUD first episodes were caused by HSV-1.^35^ Our estimate for the Americas was similar, with 55% of first episodes due to HSV-1. Such trends may be occurring elsewhere in the world.^97^ Although the potential for genital HSV-1 infection postchildhood is uncertain, our analyses suggest that genital HSV-1 only makes a small contribution to all GUD globally, given the vastly greater number of HSV-2 recurrences.

Similarly, a third limitation concerns the availability, quality and representativeness of natural history data. Studies differed on a number of characteristics, including population group (women or men or both, or men who have sex with men), study location, method of identifying lesions (eg, clinician vs self-report) and length of follow-up. Pooling data from these disparate studies may have introduced bias in our pooled parameter estimates; however, if we excluded more studies, we would have further reduced the availability of data, particularly for settings outside of the USA. The vast majority of the data on first GUD episodes and recurrences, which informed our natural history parameters, came from studies in the USA, which may not reflect the natural history of HSV infection elsewhere. To help mitigate some of these issues, we applied specific inclusion and exclusion criteria for data extraction, pooled data from similar populations as far as possible (ie, those with diagnosed vs unrecognised infection), and standardised data prior to pooling (ie, converting medians to means, and calculating annualised recurrence frequencies).

In addition, the natural history data did not always align perfectly with the possible states for GUD. Our estimate that 4.8% of the world’s population had at least one episode of HSV-2-related GUD globally in 2016 is equivalent to 36% of those with prevalent HSV-2 infection. We would expect this figure not to exceed the percentage with a first episode (since by our definition, only those with a first episode can experience subsequent recurrences). Our pooled estimate of the percentage with a first episode is somewhat below this figure (21.0%). However, not all of the studies contributing data to this estimate were rigorously designed to ensure all those with GUD were identified: one study relied on self-reported symptoms, for example,^37^ while another only considered the 6 months prior to seroconversion.^38^ Indeed, a rigorous clinical trial of GUD associated with seroconversion found that 36% had symptoms.^36^ In addition, natural history studies may enrol people with more severe infection, leading to overestimates of the number and duration of recurrences. In our base case estimates, we used the method that we felt most closely aligned available data with the possible clinical courses for GUD. Our sensitivity analysis showed that the estimates were sensitive to the relative percentages that recurrence rates from clinic-based studies and studies of unrecognised infection are applied to. Therefore, new studies of the natural history among all those with HSV-2 infection would be useful.

Fourth, we assumed that both the percentage who experience one or more recurrences in a given year after the first year and the duration of a recurrence are independent of time since infection (generating one pooled recurrence duration estimate using data with any time since infection), although we did allow the recurrence rate to vary over time. The one available study which examined recurrence frequency and duration over a wide range of time since infection found no change in recurrence frequency but a small decline in recurrence duration, leading to an overall decline in the percentage of days with GUD.^64^ Our pooled parameter estimate for the mean number of days with recurrent GUD due to HSV-2 was actually slightly increased for longer time since infection (although 95% CI had a large overlap), perhaps because of the disparate studies combined, or perhaps because we did not account for decreasing recurrence duration over time. Therefore we may have overestimated GUD burden, as our sensitivity analysis showed that the burden would be lower if recurrences stopped after more than 10 years since acquisition of HSV-2 infection. However, we also did not consider GUD burden in those aged 50 years or over, potentially leading to an underestimation of burden overall rather than an overestimation. We estimated that individuals with HSV-2 infection experience on average 16 days with GUD annually. Recurrence data from a study excluded from the pooling because it was among all those who are HSV-2 seropositive (including those who were asymptomatic) found a median number of annual recurrences of 2.1,^98^ which when applied to estimates of the mean recurrence duration for those who are HSV-2 seropositive (data also unused), which ranged from 7 to 10 days,^99–101^ gives an estimate of 15–21 days with GUD annually, which is similar to our estimate of 16 days.

Finally, we did not consider the modifying effect of previous HSV-1 infection on GUD due to HSV-2. Prior studies have shown that existing HSV-1 infection has no effect on subsequent HSV-2 acquisition,^102^ but that those with pre-existing HSV-1 infection are more likely to have asymptomatic HSV-2 acquisition compared with those who are HSV-1 seronegative.^9^ The recurrence rate is similar among symptomatic people with and without HSV-1 infection.^42 54^ For the purposes of this exercise, we assumed an absence of interactions between HSV-1 and HSV-2, which may have led to a slight overestimation of GUD among those with coinfection. Given that the number of people affected by GUD due to genital HSV-1 is small relative to HSV-2, we also assumed that total GUD burden is simply the sum of GUD burden for HSV-2 and GUD burden for genital HSV-1. We further assumed that among HSV-2 seropositive persons, all GUD was related to HSV-2 infection. Some studies of HSV-2 recurrence rate may have inadvertently captured some GUD due to genital HSV-1. However, given that studies specific to HSV-1 found low genital HSV-1 recurrence rates, and HSV-1 infection only rarely follows HSV-2,^9^ this was unlikely to have been a significant limitation. We further assumed no contribution to GUD from non-HSV aetiologies in those with genital HSV infection. The inclusion of non-HSV-attributable GUD could potentially have led to an overestimation of GUD due to HSV, but this is unlikely to have been a major issue given the types of studies contributing data, many of which were rigorous clinical trials.

## Conclusions

Up to 187 million people aged 15–49 years may experience HSV-related GUD annually, with women disproportionately affected, and the highest burden of GUD in Africa. This large burden is a public health concern in itself, but especially so since HIV and GUD are similarly distributed across populations, maximising the biological potential for GUD to increase both susceptibility to, and transmissibility of, HIV.^13 15 16^

Our estimates do not provide insight into the severity of symptoms beyond frequency and duration of recurrences. GUD can have a substantial impact on the lives of those it affects, not only in terms of physical pain and discomfort, but also psychosocially.^8 103^ At the same time, many people with HSV GUD do not recognise they have herpes and never seek care for their symptoms. Better data are needed on how the GUD burden estimated here translates into overall impact on quality of life in terms of quality-adjusted or disability-adjusted life years.^104^ In the mean time, the substantial burden of GUD can be ameliorated by antivirals, which are not widely used worldwide for HSV-related GUD. Accessibility to antivirals, along with accurate diagnostics, therefore needs to be increased in order to improve the lives of the millions of people with GUD globally.^93^ Our estimates also show that new interventions such as prophylactic or therapeutic HSV vaccines, new antivirals that can suppress the virus, or microbicides may have a large public health potential to reduce GUD both by reducing the frequency of symptoms in the millions of individuals who already have GUD and also perhaps by having a meaningful impact on the transmission of infection and subsequent GUD.
