# Supplementary material for: The global and regional burden of genital ulcer disease due to herpes simplex virus: a natural history modelling study
Source: BMJ Glob Health. 2020 Mar 8;5(3):e001875. doi: 10.1136/bmjgh-2019-001875 (PMC7061890; doi:10.1136/bmjgh-2019-001875)
Supplement: Supplementary data [file bmjgh-2019-001875supp001.pdf]

Appendix: Global and regional estimates of genital ulcer disease due to herpes simplex virus

Dr Katharine J. Looker, Professor Christine Johnston, Professor Nicky J. Welton, Dr Charlotte James, Professor Peter Vickerman, Dr Katherine M. E. Turner, Professor Marie-Claude Boily and Dr Sami L. Gottlieb

FURTHER DETAILS ON THE METHODS

Natural history model

GUD due to HSV-2 infection

The percentage of people with at least one episode of GUD due to HSV-2 in a given year at age *a* is a function of:

- The percentage of individuals with recently-acquired infection (τ≤1 year since infection) who have a first episode of GUD (P_{first}), multiplied by the percentage of the population with recently-acquired HSV-2 infection, $I(a)$ (among all individuals, not just those with HSV-2 infection);
- The percentage of individuals with established infection (τ>1 year since infection) who have one or more GUD recurrences in a year after the first year of infection, $P_{recur_{\tau>1}}$, multiplied by the percentage of the population with established HSV-2 infection, $F(a)$.

The equation is as follows:

Percentage with any GUD due to HSV-2 in a given year at age *a* (expressed as a percentage of the total population) =

$\left[I(a) * P_{first}\right] + \left[F(a) * P_{recur_{\tau>1}}\right]$ (equation i).

P_{first} is measured by prospective studies (often clinical trials) that evaluate GUD symptoms typically during one year of follow-up among those who seroconverted to HSV-2 during the study (**Table A1; Figure 1**).

$P_{recur_{\tau>1}}$ is informed by clinic-based studies that recruit individuals with a (diagnosed) first episode due to HSV-2 and measure the percentage of individuals with at least one recurrence during follow-up (**Table A1; Figure 1**). It is also informed by studies of unrecognised infection which recruit individuals who are HSV-2 seropositive but without a history of (recognised) genital herpes and observe how many experience documented GUD during follow-up. Deriving $P_{recur_{\tau>1}}$ estimates from these data is difficult because: (1) the percentage of infected people who have diagnosed versus unrecognised infection is difficult to define and measure, and may depend on factors such as access to healthcare; (2) these two different types of study population are not necessarily exhaustive of all those with HSV-2 infection: clinic-based studies may disproportionately include infected individuals with more severe disease, meaning that the percentage who recur and the recurrence rate measured in these studies may be overestimates; (3) GUD in those with unrecognised/undiagnosed infection makes an important contribution to disease burden.

Thus, for our base case estimates we calculated the second part of the equation by applying estimates of the percentage of individuals with established infection (τ>1 year since infection) who have received a diagnosis, $\theta = 1$, who have one or more GUD recurrences in a year after the first year of infection as measured by clinic studies, denoted by $P_{recur_{\tau>1,\theta=1}}$, to estimates of the percentage with diagnosed infection, $P_{\theta=1}$, and additionally considering the percentage with undiagnosed infection, $P_{\theta=0} = 1 - P_{\theta=1}$, who have one or more GUD recurrences as measured by studies of those with unrecognised infection, denoted by $P_{recur_{\tau>1,\theta=0}}$ (equation ii):

$\left[I(a) * P_{first}\right] + \left[F(a) * ([P_{\theta=1} * P_{recur_{\tau>1,\theta=1}}] + [P_{\theta=0} * P_{recur_{\tau>1,\theta=0}}])\right]$ (equation ii).

$P_{\theta=1}$ is informed by surveys of all those HSV-2 infected which ask participants if they have received a diagnosis (**Table A1**). We assumed that the percentage of individuals with established infection who experience recurrences is independent of time since infection ($P_{recur_{\tau>1}} = P_{recur_{\tau}}$), given the lack of data to fully inform how this might change over time. This does not necessarily mean that the recurrence *rate* does not vary with time since infection.

The mean number of days with GUD due to HSV-2 in a given year depends on the contribution to GUD days from individuals with recently-acquired infection experiencing their first episode (in the first year since infection), from individuals with recently-acquired infection with recurrences (in the first year since infection), and from individuals with established infection with recurrences (after the first year since infection), i.e.:

1. The number of GUD days per person with recently-acquired infection (τ≤1) experiencing a first episode, $T_{first_{\tau\leq1}}$, multiplied by the percentage of the population with recently-acquired HSV-2 infection, $I(a)$, and the percentage of individuals with recently-acquired infection who experience a first episode, P_{first};
2. The number of GUD days per person with recently-acquired infection (τ≤1) due to all recurrences in the first year, $T_{recur_{\tau\leq1}}$ (averaged over all those with recently-acquired infection, including those without a first episode or recurrences), among diagnosed individuals, $\theta = 1$, and undiagnosed individuals, $\theta = 0$, multiplied by the percentage of the population with recently-acquired HSV-2 infection, $I(a)$;
3. The number of GUD days per person 1<τ≤10 years following infection due to all recurrences in a year, $T_{recur_{1<\tau\leq10}}$ (averaged over all those with established infection, including those without a first episode or recurrences), among diagnosed individuals, $\theta = 1$, and undiagnosed individuals, $\theta = 0$, multiplied by the percentage of the population with established HSV-2 infection, $F(a)$;
4. The number of GUD days per person τ>10 years following infection due to all recurrences in a year, $T_{recur_{\tau>10}}$ (averaged over all those with established infection, including those without a first episode or recurrences), among diagnosed individuals, $\theta = 1$, and undiagnosed individuals, $\theta = 0$, multiplied by the percentage of the population with established HSV-2 infection, $F(a)$;

The equation is therefore as follows:

Mean number of days with GUD due to HSV-2 in a given year at age *a* =

$\left[I(a) * P_{first} * T_{first_{\tau\leq1}}\right] + \left[I(a) * ([P_{\theta=1} * T_{recur_{\tau\leq1,\theta=1}}] + [P_{\theta=0} * T_{recur_{\tau\leq1,\theta=0}}])\right] + \left[\sum_{x=1}^{x=9} I(a-x) * ([P_{\theta=1} * T_{recur_{1<\tau\leq10,\theta=1}}] + [P_{\theta=0} * T_{recur_{1<\tau\leq1\ ,\theta=0}}])\right] + \left[\sum_{x=10}^{x=a-15} I(a-x) * ([P_{\theta=1} * T_{recur_{\tau>10,\theta=1}}] + [P_{\theta=0} * T_{recur_{\tau>10,\theta=0}}])\right]$ (equation iii);

Studies may measure the percentage of days on which individuals experience GUD recurrences, which when multiplied by the total number of days in a year gives $T_{recur_{\tau}}$. Studies may also measure the

average duration of a recurrence, D_{recur}, and the number of recurrences in a year, $N_{recur_{\tau}}$, which when multiplied together also give $T_{recur_{\tau}}$.

$T_{first_{\tau\leq1}}$ is measured by studies of documented first-episode symptoms due to HSV-2 (**Table A1; Figure 1**). Similar to estimates for the percentage of people with any GUD due to HSV-2 in a given year, we calculated the latter parts of the equation by applying estimates of $T_{recur_{\tau}}$ among those who have received a diagnosis, $T_{recur_{\tau,\theta=1}}$, as measured by clinic studies, to estimates of $P_{\theta=1}$, and applying estimates of $T_{recur_{\tau}}$ among those who have not received a diagnosis, $T_{recur_{\tau,\theta=1}}$, as measured by studies of unrecognised infection, to estimates of $P_{\theta=1}$. Since $T_{recur_{\tau,\theta=0}}$ is informed by studies in those with unknown time since infection (as infection is unrecognised), $T_{recur_{\tau,\theta=0}}$ cannot be potentially assigned different values according to time since infection, meaning $T_{recur_{\tau\leq1,\theta=0}}$=$T_{recur_{1<\tau\leq1\ ,\theta=0}}$=$T_{recur_{\tau>10,\theta=0}}$.

GUD due to genital HSV-1 infection

For genital HSV-1, recurrences were limited to the first five years since infection, because there is a low recurrence rate up to five years since infection and no data available past 5 years. P_{firs} , $T_{first_{\tau\leq1}}$, $P_{recur_{\tau>1}}$and $T_{recur_{\tau}}$ (**Table A1; Figure 1**) are informed by similar studies to those for HSV-2, with the difference that there are no studies of recurrences in those with unrecognised infection, and the percentage who are diagnosed is unknown: only P_{first} can be estimated.

The relevant equations for the percentage with any GUD due to genital HSV-1 in a given year at age *a* (expressed as a percentage of the total population) and the mean number of days with GUD due to genital HSV-1 in a given year at age *a* are as follows:

$\left[I(a) * P_{first}\right] + \left[\sum_{x=1}^{x=4} I(a-x) * P_{first} * P_{recur_{\tau>1|first}}\right]$ (equation iv);

$\left[I(a) * P_{first} * T_{first_{\tau\leq1}}\right] + \left[I(a) * P_{first} * T_{recur_{\tau\leq1|first}}\right] + [\sum_{x=1}^{x=4} I(a-x) * P_{first} * T_{recur_{\tau>1|first}}]$ (equation v);

where:

$P_{recur_{\tau>1|first}}$is the percentage of individuals with established infection (τ>1 year since infection) and who had a first episode who have one or more GUD recurrences in a year after the first year of infection;

$T_{recur_{\tau\leq1|first}}$is the number of GUD days per person with recently-acquired infection (τ≤1) due to all recurrences in the first year among those who had a first episode;

$T_{recur_{\tau>1|first}}$ is the number of GUD days per person τ>1 following infection due to all recurrences in a year among those who had a first episode.

The number of people with any GUD over the total population was calculated by multiplying the percentage with GUD from equations ii and iv by total population size, $N(a)$. Person-days with GUD per total population was calculated by multiplying mean number of days with GUD from equations iii and v by total population size, $N(a)$. Using all the information we had, we also calculated the contribution to GUD burden of established versus recent infection, the total number of first episodes and the total number of recurrences, and the number of GUD days per person with infection and per person experiencing GUD.

Data sources for model parameters

$I(a)$ and $F(a)$ were taken from our existing WHO global and regional estimates of HSV-2 and genital HSV-1 infection annual incidence, $I(a)$, and prevalence, $F(a)$, in 2016 among those aged 15-49 years expressed as percentages of the population[2]. These estimates were done for 2016 informed by comprehensive literature reviews conducted up to August 2018. Full details are reported in the corresponding paper[2]. Of note, the estimates for genital HSV-1 infection are particularly uncertain, due to a lack of HSV-1 prevalence data among children for all regions, and in general for WHO Africa and South-East Asia regions in particular, and use of a pooled estimate for the percentage of incident HSV-1 infection from age 15 years that is genital, $P_{genital}$, to determine $I(a)$ and $F(a)$ for genital HSV-1 infection, informed by only four available longitudinal studies (all from the US). Population size, $N(a)$, was obtained from the United Nations Population Division[28].

Natural history parameters were informed by a PubMed literature search of English-language titles reporting on longitudinal studies (date of search 06/11/2017) using the following search terms: [ulcers (title/abstract) OR lesions (title/abstract) OR vesicles (title/abstract) OR first episode (title/abstract) OR first-episode (title/abstract) OR recurrence (title/abstract) OR disease (title/abstract) OR GUD (title/abstract) OR symptoms (title/abstract)] AND [HSV2 (title/abstract) OR HSV1 (title/abstract) OR HSV-2 (title/abstract) OR HSV-1 (title/abstract) OR herpes simplex (title/abstract) OR herpes virus type 2 (title/abstract) OR herpes virus type 1 (title/abstract) OR herpes virus 2 (title/abstract) OR herpes virus 1 (title/abstract) OR herpesvirus 2 (title/abstract) OR herpesvirus 1 (title/abstract) OR genital herpes (title/abstract)] AND (cohort analysis[MeSH Terms] OR randomized controlled trial[Publication Type] OR viral shedding[MeSH Terms]) AND genital (title/abstract); limits: humans.

Natural history parameter data were limited to GUD and not any other symptoms such as dysuria or fever. We extracted data separately for HIV-negative populations, and PLHIV or populations stated to include those HIV-positive. For the latter, we additionally extracted information on HIV prevalence and antiretroviral (ART) use. However, only data from HIV-negative populations were subsequently used in the estimates. Where it was possible to identify when multiple publications had reported on the same natural history parameter from the same study population, we extracted all data but only used the value associated with the largest sample size to inform the parameter in question.

Studies had to report GUD natural history for either HSV-2 or genital HSV-1, except for the duration of a first episode of GUD, T_{first}, which is clinically indistinguishable between HSV-2 and genital HSV-1[6]. T_{first} estimates for HSV-2 pooled together data on first episodes due to HSV-2 or either HSV type (data not separable by type), while T_{first} estimates for genital HSV-1 pooled together data on first episodes due to HSV-1 or either HSV type (data not separable by type), but for the latter, excluding data from those with existing HSV-1 infection (non-primary HSV-2 infection) where possible. Apart from this, we did not consider the effect of sex or pre-existing HSV-1 infection (for the HSV-2 GUD estimates) on the assumed parameter values. For both HSV-2 and genital HSV-1, we took the mean value where results were only reported separately for each sex, and for HSV-2, we took the mean value where results were only reported separately for primary and non-primary infection. Recurrences in those HSV-2 seropositive were assumed to be due to HSV-2. To minimize overestimating GUD, we excluded parameters from any study which selected participants based on having 4 recurrences or more per year, except for recurrence duration. Only data from the placebo/control group were extracted from clinical trials. Based on the available data, it was decided to separately pool D_{recur} and N_{recur_τ}, deriving N_{recur_τ} from T_{recur_τ} using our pooled estimate for D_{recur} where data were available for T_{recur_τ} and not N_{recur_τ}. It was decided *a priori* to allow N_{recur_τ} to vary by time since infection but

not D_{recur}, as we considered N_{recur_τ} most likely to change over time, and to maximise the number of estimates available for pooling for D_{recur}.

Pooling of studies reporting binary outcomes

Studies with reporting binary outcomes (P_{first}, $P_{recur_{\tau>1}}$ and $P_{\theta=1}$) were pooled on the log odds scale, where the standard error (SE) of the log odds was calculated according to SE=sqrt((1/cases)+(1/noncases)). This was computed using the metan command in Stata 13.1.

Pooling of studies reporting duration of symptoms

For studies reporting duration of symptoms ($T_{first_{\tau\leq1}}$ and D_{recur_τ}), we pooled mean duration on the log scale. For studies reporting median duration we estimated the mean by assuming an underlying Exponential distribution for duration of symptoms, using the formula mean=median/ln(2). SE of the mean duration was calculated as SE=mean/sqrt(N), also based on an underlying Exponential distribution for duration of symptoms. Mean duration was pooled on the log scale, which was achieved by transforming the mean and SE from a natural scale to a log scale, using the formulae from a log-Normal distribution: the log mean duration calculated from ln(mean/(sqrt(1+(SE/mean)^2))) and SE of the log mean duration calculated from sqrt(ln(1+(SE/mean)^2)). These were then pooled using the metan command in Stata 13.1, noting that the resulting pooled estimates were on the log-scale.

Pooling of studies reporting event counts

For studies reporting event counts (i.e., frequency of recurrences, N_{recur_τ}), we pooled mean event counts on the log scale. For studies reporting median event counts, we estimated the mean using the formula mean=((median-1/3)+sqrt(((1/3)-median)^2+0.08))/2, which is based on an underlying Poisson distribution for event frequency. SE of the mean frequency was calculated as SE=sqrt(mean/N), again based on an assumed Poisson distribution for event frequency. Mean event rates were pooled on the log scale, as for duration, noting that the resulting pooled estimates were on the log scale.

Table A1 Default parameter values used in the GUD estimates.

Parameter	Symbol	Corresponding state(s) in Figure 1	Data sources	Number of contributing studies (refs)	Value (95%CI)
**HSV-2 infection**					
Percentage of individuals with recently-acquired HSV-2 infection	$I(a)$	--	WHO global and regional estimates of HSV-2 incidence among those aged 15-49 years for 2016	1 set of estimates[2]	See corresponding paper[2]
Percentage of individuals with recently-acquired infection who experience a first episode (up to one year since infection was acquired)	P_{first}	2/(2+3)	Studies prospectively following HSV-2 seronegative subjects for HSV-2 seroconversion, and then evaluating first-episode GUD symptoms among those who seroconvert	8 studies[9,32-38]	21.0% (12.7%, 32.8%), I^2=90.1%; 35.5% reported in a clinical trial with rigorous assessment of GUD[30]
Percentage of individuals with established HSV-2 infection	$F(a)$	--	WHO global and regional estimates of HSV-2 prevalence among those aged 15-49 years for 2016	1 set of estimates[2]	See corresponding paper[2]
Percentage of individuals with HSV-2 infection that have been diagnosed with genital herpes	$P_{\theta=1}$	--	National Health and Nutrition Examination Survey (NHANES) 2007-2010 USA population-based evaluation of the percentage of those HSV-2 infected who are diagnosed	1 study[18]	12.6% (9.7%, 16.3%)
Percentage of individuals with established infection who have one or more GUD recurrences in a year (more than one year since infection was acquired)	$P_{recur_{\tau>1}}$	7/(7+8+9+10)	$P_{recur_{\tau>1,\theta=1}}$		
			Studies of GUD recurrences due to HSV-2 following a documented first episode ("clinic-based studies")	6 studies[7,39-43]	83.8% (69.5%, 92.2%), I^2=89.7%
			$P_{recur_{\tau>1,\theta=0}}$		
			Studies of GUD symptoms due to HSV-2 among those found to be HSV-2 seropositive but without a history of recognised GUD	3 studies[44-46]	28.1% (13.6%, 49.3%), I^2=78.5%
Mean number of GUD days per person with recently-acquired infection and experiencing a first episode	$T_{first_{\tau\leq1}}$	2	Studies of documented first-episode GUD symptoms due to genital HSV-1 or HSV-2	14 studies[40,47-59]	20.5 days (18.3 days, 22.8 days), I^2=40.3%
Mean annual number of GUD days due to recurrences per person with recently-acquired infection (up to one year since infection was acquired)	$T_{recur_{\tau\leq1}}$	4 (rate includes those without recurrences)	$T_{recur_{\tau\leq1,\theta=1}}$		
			Studies of GUD recurrences due to HSV-2 among those with recently-acquired infection who have had a documented first episode or who otherwise have a history of GUD ("clinic-based studies")	Derived from the pooled mean annual number of recurrences, $N_{recur_{\tau\leq1,,\theta=1}}$, 4.6 (95%CI 3.9, 5.5), I^2=96.2% based on 10 studies[29,40,42,60-66] and pooled mean recurrence duration, $D_{recur_{\theta=1}}$, 8.5 (95%CI 7.5, 9.5), I^2=75.8% based on 13 studies[54,64,66-76] using the equation $N_{recur_{1\leq\tau,\theta=1}} * D_{recur_{\theta=1}}$	10.7% * 365

			$T_{recur_{\tau\leq1,\theta=0}}$		
			Studies of GUD symptoms due to HSV-2 among those found to be HSV-2 seropositive but without a history of recognised GUD (unknown time since infection by definition)	Derived from the pooled mean annual number of recurrences, $N_{recur_{\tau\leq1,\theta=0}}$, 3.4 (95%CI 2.7, 4.4), I^2=88.8% based on 5 studies[34 44 45 67 77] and pooled mean recurrence duration, $D_{recur_{\theta=0}}$, 3.0 (95%CI 1.6, 5.7) based on 1 study[67] using the equation $N_{recur_{\tau\leq1,\theta=0}} * D_{recur_{\theta=0}}$	2.8% * 365
Mean annual number of GUD days due to recurrences per person with established infection (more than one year since infection was acquired)	$T_{recur_{\tau>1}}$	7 (rate includes those without recurrences)	$T_{recur_{1<\tau\leq10,\theta=1}}$		
			Studies of GUD recurrences due to HSV-2 among those with established infection who have had a documented first episode or who otherwise have a history of GUD ("clinic-based studies")	Derived from the pooled mean annual number of recurrences, $N_{recur_{1<\tau\leq10,\theta=1}}$, 5.2 (95%CI 4.1, 6.5), I^2=98.7% based on 10 studies[44 46 60 63 64 77-81] and pooled mean recurrence duration, $D_{recur_{\theta=1}}$, 8.5 (95%CI 7.5, 9.5), I^2=75.8% based on 13 studies[54 64 66-76] using the equation $N_{recur_{1<\tau\leq10,\theta=1}} * D_{recur_{\theta=1}}$	12.0% * 365
			$T_{recur_{\tau>10,,\theta=1}}$		
			Studies of GUD recurrences due to HSV-2 among those with established infection who have had a documented first episode or who otherwise have a history of GUD ("clinic-based studies")	Derived from the pooled mean annual number of recurrences, $N_{recur_{\tau>10,\theta=1}}$, 7.1 (95%CI 3.9, 13.0), I^2= 98.2% based on 2 studies[64 82] and pooled mean recurrence duration, $D_{recur_{\theta=1}}$, 8.5 (95%CI 7.5, 9.5), I^2=75.8% based on 13 studies[54 64 66-76] using the equation $N_{recur_{\tau>10,\theta=1}} * D_{recur_{\theta=1}}$	16.4% * 365
			$T_{recur_{\tau>1,\theta=0}}$		

			Studies of GUD symptoms due to HSV-2 among those found to be HSV-2 seropositive but without a history of recognised GUD (unknown time since infection by definition)	Derived from the pooled mean annual number of recurrences, $N_{recur_{\tau>1,\theta=0}}$, 3.4 (95%CI 2.7, 4.4), I^2=88.8% based on 5 studies[34 44 45 67 77] and pooled mean recurrence duration, $D_{recur_{\theta=0}}$, 3.0 (95%CI 1.6, 5.7) based on 1 study[67] using the equation $N_{recur_{\tau>1,\theta=0}} * D_{recur_{\theta=0}}$	2.8% * 365
**Genital HSV-1 infection**					
Percentage of individuals with recently-acquired genital HSV-1 infection	$I(a)$	--	WHO global and regional estimates of genital HSV-1 incidence among those aged 15-49 years for 2016	1 set of estimates[2]	See corresponding paper[2]
Percentage of individuals with recently-acquired infection who experience a first episode (up to one year since infection was acquired)	P_{first}	2/(2+3)	Studies prospectively following HSV-1 seronegative subjects for genital HSV-1 seroconversion, and then evaluating first-episode GUD symptoms among those who seroconvert	5 studies[9 33 35 36 83]	34.8% (26.7%, 44.0%), I^2=0.0%
Percentage of individuals with established genital HSV-1 infection	$F(a)$	--	WHO global and regional estimates of genital HSV-1 prevalence among those aged 15-49 years for 2016	1 set of estimates[2]	See corresponding paper[2]
Percentage of individuals with established infection who have one or more GUD recurrences in a year (more than one year since infection was acquired)	$P_{recur_{\tau>1}}$	7/(7+8+9+10)	Studies of GUD recurrences due to genital HSV-1 following a documented first episode ("clinic-based studies")	4 studies[36 40 42 84]	47.3% (31.5%, 63.6%), I^2=72.5%
Mean number of GUD days per person with recently-acquired infection and experiencing a first episode	$T_{first_{\tau\leq1}}$	2	Studies of documented first-episode GUD symptoms due to genital HSV-1 or HSV-2	14 studies [40 47-59]	20.8 days (18.6 days, 23.3 days), I^2=28.2%
Mean annual number of GUD days due to recurrences per person with recently-acquired infection (up to one year since infection was acquired) who had a first episode	$T_{recur_{\tau\leq1\|first}}$	4 (rate includes those without recurrences)	Studies of GUD recurrences due to genital HSV-1 among those with recently-acquired infection who have had a documented first episode ("clinic-based studies")	$T_{recur_{\tau\leq1\|first}}$ derived from the pooled mean annual number of recurrences, $N_{recur_{\tau\leq1}}$, 0.7 (95%CI 0.5, 1.2), I^2=89.7% based on 6 studies[7 29 40 42 61 84] and pooled mean recurrence duration, D_{recur}, 10.1 (95%CI 7.9, 12.7) based on 1 study[84] using the equation $N_{recur_{\tau\leq1}} * D_{recur}$	2.0% * 365

Mean annual number of GUD days due to recurrences per person with established infection (more than one year since infection was acquired) who had a first episode	$T_{recur_{\tau>1\|first}}$	7 (rate includes those without recurrences)	Studies of GUD recurrences due to genital HSV-1 among those with established infection who have had a documented first episode ("clinic-based studies")	$T_{recur_{\tau>1\|first}}$ derived from the pooled mean annual number of recurrences, $N_{recur_{\tau>1}}$, 0.003 (95%CI 0.0, 2.5), I^2=94.9% based on 3 studies[63 81 84] and pooled mean recurrence duration, D_{recur}, 10.1 (95%CI 7.9, 12.7) based on 1 study[84] using the equation $N_{recur_{\tau>1}} * D_{recur}$	0.008% * 365

For a full explanation of symbols see main text. 95%CI: 95% confidence interval. I^2: percentage of variation in estimate explained by between-study variation.

GATHER checklist

GATHER
Guidelines for Accurate and Transparent Health Estimates Reporting

Item #	Checklist item	Reported on page #
**Objectives and funding**		
1	Define the indicator(s), populations (including age, sex, and geographic entities), and time period(s) for which estimates were made.	Introduction para 3
2	List the funding sources for the work.	Funding section
**Data Inputs**		
For all data inputs from multiple sources that are synthesized as part of the study:		
3	Describe how the data were identified and how the data were accessed.	Literature search and pooling section, and Data sources for model parameters section in the Appendix
4	Specify the inclusion and exclusion criteria. Identify all ad-hoc exclusions.	
5	Provide information on all included data sources and their main characteristics. For each data source used, report reference information or contact name/institution, population represented, data collection method, year(s) of data collection, sex and age range, diagnostic criteria or measurement method, and sample size, as relevant.	Table A1
6	Identify and describe any categories of input data that have potentially important biases (e.g., based on characteristics listed in item 5).	Table A1 and Discussion
For data inputs that contribute to the analysis but were not synthesized as part of the study:		
7	Describe and give sources for any other data inputs.	Methods, and Data sources for model parameters section in the Appendix
For all data inputs:		
8	Provide all data inputs in a file format from which data can be efficiently extracted (e.g., a spreadsheet rather than a PDF), including all relevant meta-data listed in item 5. For any data inputs that cannot be shared because of ethical or legal reasons, such as third-party ownership, provide a contact name or the name of the institution that retains the right to the data.	Table A1
**Data analysis**		
9	Provide a conceptual overview of the data analysis method. A diagram may be helpful.	Methods, Figure 1, and Natural history model section in the Appendix
10	Provide a detailed description of all steps of the analysis, including mathematical formulae. This description should cover, as relevant, data cleaning, data pre-processing, data adjustments and weighting of data sources, and mathematical or statistical model(s).	Methods, and Natural history model and Data sources for model parameters sections in the Appendix
11	Describe how candidate models were evaluated and how the final model(s) were selected.	Methods, and Natural history

		model section in the Appendix
12	Provide the results of an evaluation of model performance, if done, as well as the results of any relevant sensitivity analysis.	Sensitivity analysis section in the Results
13	Describe methods for calculating uncertainty of the estimates. State which sources of uncertainty were, and were not, accounted for in the uncertainty analysis.	Uncertainty analysis section in the Methods
14	State how analytic or statistical source code used to generate estimates can be accessed.	N/A
Results and Discussion		
15	Provide published estimates in a file format from which data can be efficiently extracted.	Tables 1-3 and Appendix
16	Report a quantitative measure of the uncertainty of the estimates (e.g. uncertainty intervals).	Table 3
17	Interpret results in light of existing evidence. If updating a previous set of estimates, describe the reasons for changes in estimates.	Results and Discussion
18	Discuss limitations of the estimates. Include a discussion of any modelling assumptions or data limitations that affect interpretation of the estimates.	Discussion

Blank checklist downloaded from gather-statement.org[30 31]

FURTHER RESULTS

Table A2 Global and regional estimates of the number of first episodes and the number of recurrences of GUD among the total population (in millions) among 15-49 year olds in 2016, by HSV type, sex and WHO region

WHO region	Sex	Age (years)			
		No. of first episodes of GUD due to HSV-2	No. of GUD recurrences due to HSV-2	No. of first episodes of GUD due to HSV-1	No. of GUD recurrences due to HSV-1
**Americas**	**Females**	0.6	112.4	0.5	1.0
	**Males**	0.3	54.4	0.5	1.0
**Africa**	**Females**	1.0	204.1	0.0	0.0
	**Males**	0.7	115.7	0.0	0.0
**Eastern Mediterranean**	**Females**	0.1	24.8	0.2	0.4
	**Males**	0.1	9.8	0.2	0.4
**Europe**	**Females**	0.2	43.1	0.2	0.4
	**Males**	0.1	21.5	0.5	0.8
**South-East Asia**	**Females**	0.5	93.9	0.0	0.0
	**Males**	0.4	74.6	0.0	0.0
**Western Pacific**	**Females**	0.7	134.9	0.1	0.1
	**Males**	0.4	69.8	0.0	0.1
**Global**	**Females**	**3.1**	**613.2**	**1.1**	**2.0**
	**Males**	**1.9**	**345.9**	**1.3**	**2.3**
	**Both**	**5.0**	**959.1**	**2.4**	**4.3**

Table A3 Sensitivity analysis exploring the effect on global and regional estimates of the number (in millions) and percentage of people with any GUD, and GUD person-days (in millions) among the total population, due to HSV-2 among 15-49 year olds in 2016 of (a) limiting recurrences to the first 10 years since HSV-2 infection, (b) applying recurrence natural history parameters from studies of those with unrecognised infection to all those with HSV-2 infection, (c) applying recurrence natural history parameters obtained from clinic studies to the maximum percentage with GUD symptoms as measured in a rigorous clinical trial[36] (recurrence natural history parameters from studies of those with unrecognised infection unused), and (d) applying recurrence natural history parameters obtained from clinic studies to the percentage with a first episode, and recurrence natural history parameters from studies of those with unrecognised infection to all remaining infected individuals.

WHO region	Sex	ANY GUD				GUD PERSON-DAYS			
		(a) Recurrences limited to first 10 years[1]	(b) Rates in those with unrecognised infection applied to all those with HSV-2 infection	(c) Clinic rates applied to maximum % with symptoms	(d) Clinic rates applied to a higher proportion of infected individuals	(a) Recurrences limited to first 10 years	(b) Rates in those with unrecognised infection applied to all those with HSV-2 infection	(c) Clinic rates applied to maximum % with symptoms	(d) Clinic rates applied to a higher proportion of infected individuals
**Americas**	**Females**	9.0<br>3.7%	16.8<br>7.0%	17.8<br>7.4%	23.6<br>9.8%	396	638	1149	1180
	**Males**	4.5<br>1.9%	8.2<br>3.4%	8.6<br>3.6%	11.5<br>4.7%	200	310	554	571
**Africa**	**Females**	16.8<br>7.2%	29.9<br>12.8%	31.6<br>13.5%	42.0<br>17.9%	736	1159	2081	2140
	**Males**	10.5<br>4.5%	17.4<br>7.4%	18.3<br>7.9%	24.3<br>10.4%	464	664	1172	1213
**Eastern Mediterranean**	**Females**	2.2<br>1.3%	3.7<br>2.2%	4.0<br>2.4%	5.2<br>3.1%	98	142	252	260
	**Males**	0.9<br>0.5%	1.5<br>0.8%	1.6<br>0.9%	2.1<br>1.1%	39	56	100	103
**Europe**	**Females**	3.4<br>1.6%	6.5<br>3.1%	6.8<br>3.3%	9.1<br>4.4%	149	244	441	452
	**Males**	1.7<br>0.8%	3.2<br>1.5%	3.4<br>1.6%	4.5<br>2.2%	76	122	220	226
**South-East Asia**	**Females**	8.0<br>1.6%	14.1<br>2.8%	14.9<br>3.0%	19.8<br>3.9%	354	536	955	985
	**Males**	6.4<br>1.2%	11.2<br>2.1%	11.9<br>2.2%	15.7<br>3.0%	285	426	758	783
**Western Pacific**	**Females**	10.4<br>2.2%	20.2<br>4.2%	21.3<br>4.5%	28.3<br>5.9%	459	764	1382	1416
	**Males**	5.5	10.5	11.1	14.7	245	396	714	733

		1.1%	2.1%	2.2%	2.9%				
**Global**	**Females**	**49.7**<br>**2.7%**	**91.2**<br>**5.0%**	**96.4**<br>**5.3%**	**128.0**<br>**7.0%**	**2191**	**3483**	**6259**	**6434**
	**Males**	**29.6**<br>**1.6%**	**52.0**<br>**2.7%**	**54.9**<br>**2.9%**	**72.8**<br>**3.8%**	**1310**	**1975**	**3518**	**3629**
	**Both**	**79.3**<br>**2.1%**	**143.2**<br>**3.8%**	**151.3**<br>**4.1%**	**200.8**<br>**5.4%**	**3501**	**5458**	**9777**	**10063**

[1]Percentage with at least one recurrence after the first year of infection calculated from cumulative incidence rather than prevalence.
